# When can we Kick (Some) Humans “Out of the Loop”? An Examination of the use of AI in Medical Imaging for Lumbar Spinal Stenosis

**DOI:** 10.1007/s41649-024-00290-9

**Published:** 2024-05-15

**Authors:** Kathryn Muyskens, Yonghui Ma, Jerry Menikoff, James Hallinan, Julian Savulescu

**Affiliations:** 1 Centre for Biomedical Ethics, Yong Loo Lin School of Medicine, National University of Singapore, Singapore; 2 Centre for Bioethics, Xiamen University, Xiamen, China; 3 Yong Loo Lin School of Medicine, National University of Singapore, Singapore; 4 Uehiro Centre for Practical Ethics, Faculty of Philosophy, University of Oxford, Oxford, UK

**Keywords:** AI, Radiology, Medical imaging, AI ethics, Automation

## Abstract

Artificial intelligence (AI) has attracted an increasing amount of attention, both positive and negative. Its potential applications in healthcare are indeed manifold and revolutionary, and within the realm of medical imaging and radiology (which will be the focus of this paper), significant increases in accuracy and speed, as well as significant savings in cost, stand to be gained through the adoption of this technology. Because of its novelty, a norm of keeping humans “in the loop” wherever AI mechanisms are deployed has become synonymous with good ethical practice in some circles. It has been argued that keeping humans “in the loop” is important for reasons of safety, accountability, and the maintenance of institutional trust. However, as the application of machine learning for the detection of lumbar spinal stenosis (LSS) in this paper’s case study reveals, there are some scenarios where an insistence on keeping humans in the loop (or in other words, the resistance to automation) seems unwarranted and could possibly lead us to miss out on very real and important opportunities in healthcare—particularly in low-resource settings. It is important to acknowledge these opportunity costs of resisting automation in such contexts, where better options may be unavailable. Using an AI model based on convolutional neural networks developed by a team of researchers at NUH/NUS medical school in Singapore for automated detection and classification of the lumbar spinal canal, lateral recess, and neural foraminal narrowing in an MRI scan of the spine to diagnose LSS, we will aim to demonstrate that where certain criteria hold (e.g., the AI is as accurate or better than human experts, risks are low in the event of an error, the gain in wellbeing is significant, and the task being automated is not essentially or importantly human), it is both morally permissible and even desirable to kick the humans out of the loop.

## Introduction

New innovations in the frontiers of artificial intelligence (AI) have led to increasing areas of application for this class of technologies, as well as increasing concerns about the bounds of its ethical use. AI’s potential applications in healthcare are indeed manifold and revolutionary, and within the realm of medical imaging and radiology (which will be the focus of this paper), significant increases in accuracy and speed, as well as significant savings in cost, stand to be gained through the adoption and implementation of this technology. Because of its novelty, a norm of keeping humans “in the loop” wherever AI mechanisms are deployed has become synonymous with good ethical practice in some circles. It has been argued that keeping humans in the loop is important for reasons of safety, accountability, and the maintenance of institutional trust. However, this paper will argue that there are some scenarios where an insistence on keeping humans in the loop is unwarranted and could come with significant opportunity costs. We adopt a “bottom up” approach to make our arguments. We begin with presenting a case study using an AI model based on convolutional neural networks developed by a team of researchers (including author James Hallinan of this paper) at the National University Hospital and the medical school at the National University of Singapore (NUH/NUS) for automated detection and classification of the lumbar spinal canal, lateral recess, and neural foraminal narrowing in an MRI scan of the spine to diagnose lumbar spinal stenosis (LSS). Using this case as a starting point, we will explore the possibilities generated by such technologies, one possibility being increased automation of certain medical procedures. Some radiologists have looked upon the prospect of automating a large portion of their work with fear, worrying it may make the specialty obsolete (Hayashi 2021; Chockley and Emanuel 2016). Even so, keeping radiologists busy or employed is not sufficient reason to insist on keeping them “in the loop” if the benefits of automation are to be taken seriously. Overall, the many practical benefits to the automation of these aspects of medical imaging make it worthwhile to consider both pragmatically and morally. Increasing the efficiency and accuracy of diagnosis can mean improving the quality of care, while also lowering healthcare costs, and improving human wellbeing. These are all important and worthy gains. We will aim to demonstrate that, contrary to the present conventional thinking, where certain criteria hold, it is both morally permissible and even desirable to kick the humans out of the loop. As we will discuss, this is especially the case in low-resource settings where medical professionals may be in short supply.

## Background

The prospect of automation is increasingly a realistic one in the field of radiology, as many scholars have noted (Mazurowski 2019; Chockley and Emanuel 2016; and others). As Mazurowski (2019) has discussed, the possibility of AI replacing large numbers of radiologists has often been dismissed by the radiology community. He cites several reasons for this dismissal, including doubt that the technology will ever match human skills, the belief that legal liability issues would be insurmountable and thus regulatory bodies like the FDA would never permit machines to replace radiologists, and that patients would never place their trust in an automated system (Mazurowski 2019). Recent years have already demonstrated otherwise. While the regulation of AI in healthcare is still being developed, and patient attitudes continue to evolve, the capabilities of AI for medical imaging have proven themselves to be more than capable. Among the more prescient in the field, Chockley and Emanuel wrote in their 2016 article, that “[m]achine learning will become a powerful force in radiology in the next 5 to 10 years and could end radiology as a thriving specialty.”

More recently, Hayashi (2021) has expressed concern about automation leading to a devaluation of radiologists themselves. Writing in his review of the Spine AI case study that is the focus of this paper itself, Hayashi observes that this kind of program’s many benefits (like reduction in reading time, reduction in intrareader and interreader variabilities, and lowering costs) could lead non-radiologists to question the need for the specialty in the first place (Hayashi 2021). Indeed, an earlier study by Liu et al. (2019) found that the diagnostic performance of deep learning (DL) models in medical imaging was equivalent to that of healthcare professionals. Thus, it would seem that the day that some radiologists thought would not come is fast approaching if not dawning already. In light of this, there is a need to articulate what criteria, if any, would ever make it morally permissible to kick humans out of the loop.

We will adopt a working definition of AI from Richardson et al. (2020), and “deem a computer to exhibit artificial intelligence (AI) when it performs a task that would normally require intelligent action by a human”. The specific kind of AI employed in the present case study performs one of the tasks otherwise done by radiologists—namely, reading and measuring MRI scans of the spine.

As Marcin Rzadeczka (2020) writes, AI is being applied to nearly every aspect of medicine, in the data analysis of scientific literature, the diagnostic process, and now through the use of large language models (LLMs), AI is even capable of mediating the communication between physicians and patients. Medical imaging has been one of the areas of medical application that has garnered the most excitement in recent years (see Richardson et al. 2020; Siefert et al. 2021, among others). AI can and has been used to enhance many aspects of medical imaging, including making the process faster and more accurate. Given the repetitiveness and time-consuming nature of reading the MRI scans, this is an attractive area for the application of AI to transform radiology practice. Indeed, while most in the field have argued that the role of the radiologist will only be augmented, not replaced by AI models, others have speculated that as the capabilities of the technology improve this may change (see Jha and Topol 2016; Chockley and Emanuel 2016, and Mazurowski 2019). It is this latter possibility that we seek to explore in the following sections of this paper.

The details of the case study and its findings are as follows:

Spine AI: Medical Imaging for Lumbar Spinal Stenosis

*Developed from:* Hallinan et al. (2021)

A team of researchers at NUH/NUS medical school in Singapore (including author James Hallinan of this piece) has developed an AI model based on convolutional neural networks for automated detection and classification of the lumbar spinal canal, lateral recess, and neural foraminal narrowing in an MRI scan of the spine to diagnose lumbar spinal stenosis (LSS). LSS is a potentially debilitating condition affecting many adults globally, with a considerable impact on livelihood. Most patients with LSS present with lower back pain, which is also the main reason for seeking care. A large proportion of patients eventually undergo lumbar spine MRI for diagnosis and treatment planning. Lumbar spine MRI is an essential tool in the assessment of LSS for the accurate evaluation of the central canal, lateral recesses, and neural foramina. The degree of stenosis at each region plays a role in determining the appropriate treatment, but detailing such information in a report can be repetitive and time-consuming. In addition, there are multiple grading systems for LSS, with a lack of standardization.

The research team was able to show that the Spine AI model for *semi*-automated (e.g., human-in-the-loop) reporting of lumbar spine MRI scans could produce the following benefits:

More *consistent* and *accurate* grading of spinal stenosis: In a trial run, Spine AI performed comparably to expert human radiologists specializing in LSS (Hallinan et al. 2021, 7). Additionally, it was found to be able to assist inexperienced readers and improve their accuracy over time (Lim et al. 2022). This can improve clinical decision-making and patient outcomes (Hallinan et al. 2021), and where institutions lack radiologist expertise, the model offers a tool to improve the accuracy of inexperienced readers, e.g., kappas^1^ for trainee and general radiologists increased from 0.6 to 0.9 with the model, matching the performance of a specialist spine radiologist (kappa = 0.9) (Hallinan et al. 2021; Lim et al. 2022)

Improved scan *turnaround time* and radiologist *productivity* (which in turn *reduced cost*): The deep learning (DL) solution will also reduce the time taken for report generation. Based on the recent Radiology manuscript (Lim et al. 2022) reporting time could be reduced by ~70% with, compared to without DL model assistance (e.g., 10 minutes to 3 minutes with the DL model, 7-minute time saved) (Lim et al. 2022). With ~67,000 MRI lumbar spines a year performed in Singapore (~10 hospitals), a saving of 7 minutes per MRI results in ~469,000 minutes (7817 hours) saved per year in Singapore alone. The per-hour rate for radiologists is $100 SGD, meaning there is a potential cost savings of up to $780,000 SGD each year.

This version of assisted AI solution for MRI spine reporting has the radiologist at the center of the process (e.g., it was not fully automated). The AI model outputs are provided as boxes overlaid on the MRI images. These can be changed as necessary by the reporting radiologist, and once they have reviewed all the outputs a text report can be automatically generated.

The researchers at NUH/NUS recommend that the AI-assisted solution for MRI spine reporting have the radiologist at the center of the process, saying “a fully automated system is unlikely to be acceptable to either radiologists or clinicians” (Hallinan et al. 2021). AI models of the type deployed in this instance can assist with reading images and identifying patterns in the data *at a faster rate than humans alone*. However, the limitations of current AI models mean that the human radiologist cannot be removed from the process without raising some serious ethical concerns, primarily, concerning issues of *safety, reliability, and accountability*.

The authors of the study concluded, “…our deep learning (DL) model is reliable and may be used to quickly assess lumbar spinal stenosis (LSS) at MRI. In clinical practice, the diagnosis of LSS *still relies on the subjective opinion of the reporting radiologist.* Our DL model could provide semi-automated reporting *under the supervision of a radiologist* to provide more consistent and objective reporting. Further development of the DL model could involve a consensus panel of international experts to reduce any labeling errors and biases. The DL model could also be assessed for the longitudinal follow-up of LSS at MRI” (Hallinan et al. 2021, 137).

## Discussion

As can be seen above, Spine AI was implemented as a semi-automated system—meaning the human radiologist was still firmly within the loop during the case study trials. However, in what follows, we will be arguing that it would be permissible to kick the human out of the loop going forward, and by extension in other similar cases, that meet the criteria we will describe in the “Criteria for Kicking (Some) Humans Out of the Loop” section. Before we get to that point, however, it is useful to explore how many of the common concerns about AI might or might not apply in this case.

At first glance, this case is a simple example of the successful implementation of AI in radiology. The technology was safe, effective, and successfully implemented. In fact, this case is interesting precisely because of its “tameness” when compared with other, more controversial applications of AI technology. To explain why that is, we should first give some brief summaries of the common controversies.

Common areas of concern in the wider literature on AI in healthcare center are the following: (1) transparency/opacity, (2) risk/safety, (3) bias, (4) accountability or responsibility, and (5) trustworthiness. We will briefly discuss each in turn as it pertains to the present case. Though these concerns are not without grounds in many instances, there is a worrying tendency to treat them fetishistically, leading some to uncritically or dogmatically apply them to any and all instances where AI technologies might be employed. There is a need, therefore, to continually question where these issues are genuinely concerning.

Let us begin with the question of transparency. Many AIs are “black boxes.” In other words, the systems are “opaque,” meaning that for the users, it is unclear how the system arrived at its conclusions. Ferrario (2022) has argued that the epistemic opacity involved in AI technologies is a barrier to their trustworthiness, and that therefore, their use in medical practice cannot be normatively justified. Other scholars have echoed this need for transparency (see Liefgreen et al. 2023; Chan 2023; Pierce et al. 2021). Interestingly, Hatherley et al. (2022) observed that the demand for more “explainable” or “interpretable” AI systems may come at the cost of the very accuracy that makes these technologies attractive. Even so, they argue, a preference for interpretable systems is defensible on pragmatic grounds, as they assert that clinicians are more comfortable and therefore more likely to implement and adopt technology that they understand (Hatherley et al. 2022). In this instance, happily, the Spine AI system is a species of “interpretable” AI. As the Spine AI program reads the image, it will display what region of the scan it has used to determine the grading. Compared to some other forms of image reading AIs, which only label a scan as either showing the presence or absence of a given condition, Spine AI adds an additional valuable step, in that it visually displays highlighted regions which it uses to make the grading—allowing for easy interpretation and double checking. Thus, the system is less opaque than other AI image reading tools, which helps the clinicians involved in the study understand the system and reduces potential resistance to implementing it.

As far as questions of safety or risks to patients are involved, Spine AI also appears uncontroversial. The system merely reads the MRI scans and measures the various parts of the spinal canal. The system does not store the data, so there are no risks to the patients regarding its storage or use. Spine AI does not directly interact with the patient at all. Thus, the only direct impact that the system can have on a patient is in the event of misdiagnosis. Because of the patient pathway, which involves a consultation with a clinician both before and after the scan (regardless of whether the scan is read by a human radiologist or the Spine AI program alone), and because the condition itself is not a matter of life and death, the risks associated with overdiagnosis are low. In the case of a false positive, the patient is unlikely to be referred to unnecessary surgery because the clinician will still be present to holistically assess the patient’s symptoms and scans before proceeding. Even in cases where surgery for LSS is considered, it is not always the treatment of choice (see Katz et al. 2022 for a discussion of the range of treatments available for LSS and their success rates), and there are many additional steps that a patient would need to go through before surgery would be done—meaning there is a high chance of any initial error being caught. Thus, the risk to patients was low. In fact, the highest risk from overdiagnosis would likely be unnecessary spending on the additional scans. In the case of overdiagnosis, the benefits from the reduction in cost are less than there otherwise would be, but even so, the cost to the healthcare system is still likely to be lessened overall.

As to the question of bias, disparities in access, outcomes, and quality of healthcare across various social groups have been an area of concern for some time. Lumbar spinal conditions are no exception. Some studies have found racial disparities in the realm of spinal surgeries as well (though this data was within the USA) (see Mo et al. 2022). Additionally, it is known that AI systems sometimes have the ability to detect racial (and other kinds) of differences, even when such data is not provided, since differences do exist in the size and condition of spinal muscles across race, gender, and age (see Hida et al. 2021). In principle, this could lead to an increase in unjust disparities in care. While it is not necessarily morally concerning that racial differences are present, it could become a problem if this results in under-diagnosis of LSS in certain groups. This is a realistic possibility, given that disparities in AI performance across race has been detected in other settings. Crawford and Paglen (2021) have argued that automated interpretation of images is an inherently social and political act because it involves teaching a machine to classify images of human beings. This process of classification has historically been intertwined with various kinds of social biases (Crawford and Paglen 2021). Indeed, AI learns to classify images based on the input that the human designers give it, meaning that it is likely to replicate human biases (even those that are not conscious or intended). AI systems have been observed to be able to detect differences of race, gender, and age, even without those details included in the datasets. Even in otherwise innocuous seeming instances, these biases have the potential to translate into problematic outcomes if they replicate various unjust social disparities. However, in the present case, since the data used to train the system was racially heterogeneous (including Chinese, Malay, Indian, and Caucasian subjects) there is at least some good reason to trust the validity of its readings across at least these groups. More research would need to be done to test the real-world validity of Spine AI in other contexts and to observe whether any worrisome disparities emerge.

All that remains to discuss is accountability and trustworthiness. Both of these are strongly linked with the logic underpinning the norm of keeping humans in the loop, so we will discuss them together in the following section.

## To Automate or Not to Automate?

As mentioned in the outset of this paper, the phrase “humans in the loop” has become the shorthand within the literature to indicate the importance of reserving the ultimate decision-making power for any application of AI technology for humans, and resisting the temptation to outsource to machines (Rahwan 2018). There are many reasons for this, both moral and practical. We will begin with the practical. Many scholars have pointed out that fully automated use of AI models is not suitable *at this time*, because the technology has not been proven to be sufficiently adept. There are also cost-related reasons to keep the radiologist involved, given the current limitations of the technology. In prior reporting (in Lim et al. 2022), the authors (including author James Hallinan of this paper) pointed out, "...if the radiologist constantly supervises the job of an AI system, there is no reason to construct superstructures which, inexorably, will slow down the adoption of AI in MI [medical imaging],” meaning that the combination of the radiologist with the AI model is also the most cost-efficient at present. That said, it is reasonable to anticipate the day when this technology would be realistically able to be implemented in an automated fashion, and it is prudent to explore the ethical implications ahead of time, thus our arguments that follow are directed at that likely future scenario.

That said, some have argued that there are reasons to worry that fully automated medical AI could have corrosive effects on trust in the clinical context (Hatherley 2020). As Hatherley (2020) has argued, in order to protect trust in the institution of medicine, it is important that the ultimate responsibility for diagnosis lies with the radiologist/clinician. Though a system like Spine AI may be adept at pattern recognition, this does not mean that it can assess the holistic picture of the health of the patient in question. In our case, however, even if the radiologist were to be kicked out of the loop, the surgeon would remain at the center of the process—they must decide whether or not to operate, informed by the AI (with a radiologist or without) and other tests (e.g., neurological tests).

However, these practical concerns about trust and user uptake are likely to be highly contextual and also subject to change as the technology continues to improve. Attitudes about the trustworthiness of AI technologies are highly variable across culture and generation and also dependent upon the ways these technologies are presented and discussed in the wider media (Araujo et al. 2020). As Bunz and Braghieri (2022) discuss in their article, “The AI doctor will see you now: assessing the framing of AI in news coverage,” there is a tendency to discuss AI as “outperforming” human experts and this has the effect of placing it above critique and concern in the minds of the public (including clinicians), leading to a pressure to defer to it even when it might conflict with the Hippocratic oath. Indeed, there may be a tendency to “overtrust” AI rather than mistrust it (Krugel et al. 2022).

There are also some well-established moral reasons to insist on keeping humans in the loop. For instance, as Jotterand and Bosco (2020) have argued, AI technologies allow healthcare professionals to spend less time on many tasks, from administration to technology-related procedures, and this presents the risk of an ever more de-humanized medical system. They go on to argue that artificial intelligence should only be implemented *at all* in the medical context where it meets three criteria: (1) it serves human ends, (2) respects personal identity, and (3) promotes human interaction (Jotterand and Bosco 2020). The third criterion seems to be the most crucial, in that it is the deciding factor regarding whether healthcare AI will make the practice of medicine more human or further de-humanize it.

The prospect of de-humanized medicine should concern us for several reasons. First and foremost is the issue of accountability in the case of something going wrong (see Kempt et al. 2023). Tobia et al. (2021) have examined the ways in which reliance on medical AI can increase liability in medical malpractice. They state,

Our results indicate that physicians who receive advice from an AI system to provide standard care can reduce the risk of liability by accepting, rather than rejecting, that advice, all else equal. However, when an AI system recommends nonstandard care, there is no similar shielding effect of rejecting that advice and so providing standard care. The tort law system is unlikely to undermine the use of AI precision medicine tools and may even encourage the use of these tools (Tobia et al. 2021).^2^

Grote (2021) has found that various studies examining the interaction of AI systems and physicians have shown that without being able to evaluate their trustworthiness, physicians (especially novices) become over-reliant on this support—and ultimately are thus more likely to be led astray by incorrect decisions. Yet, other scholars have come to different conclusions. For instance, Lang (2022) has explored the relationship between the physician and automated “decision support systems” (AI-DSS) observing, that when the AI’s diagnostic accuracy supercedes or at least matches the performance of a human expert, healthcare administration can improve diagnostic performance by introducing AI-DSS without the unintended byproduct of a responsibility gap.

If we try to apply these critiques within the Spine AI case, we quickly encounter a mismatch. These concerns do not seem applicable in our use case, since Spine AI is not responsible for recommending any specific treatment for the patient, it is simply a measuring tool. Like any such tool, the information it provides may inform or support the decision of a clinician, but its role stops well short of advising any particular treatment plan. This highlights the importance of taking a bottom-up approach, as we intend to do in this paper. The particulars of each kind of health AI can vary so widely that broad generalizations are unlikely to accurately describe or adequately cover the true ethical landscape. Turning again to the Spine AI case, we can see that this scenario was one where the technology was found to be transparent, highly reliable, and low in risk to patients.

With regard to the question of accountability, the use of an AI like Spine AI does not meaningfully alter the existing ethical and legal requirements for care providers to follow the relevant standard of care. In this case, the surgeon or orthopedist responsible for the patient is still ultimately responsible for decisions regarding their patient’s care. Kicking the human out of the radiological loop does not affect where the final responsibility lies, even if it ends up that the AI might make some mistake (see Mello and Guha 2024 for more about liability risks in healthcare AI).

Spine AI also appears to force us to critically examine the real moral significance of “de-humanized” medicine. While automating a system like this could “de-humanize” this area of medicine in one sense—by removing or reducing the need for human radiologists—we should not automatically assume that this is ethically meaningful. If de-humanization is morally concerning because of the criteria Jotterand and Bosco (2020) laid out, then removing the role of radiologists could possibly be said to fall under the third criterion. Kicking any human out of any loop plausibly reduces human interaction. But who’s perspective matters here? If it is the patient’s perspective that matters, in this case, human interaction would not meaningfully be reduced, since radiologists often do not see or interact directly with patients at all, so a patient is unlikely to notice this form of automation. Plausibly, it may weaken any existing bond between clinicians and the radiologists with whom they regularly work, but it is far from obvious why this would be a serious or meaningful instance of “de-humanized” medicine. After all, the surgeon would still be responsible for outlining the case for surgery, based on all results of tests, and obtaining informed consent for surgery. Further, it is not obvious that patients do in fact care that their medical care is humanistic, especially when weighed with other values, like efficacy and efficiency. Thus, Jotterand and Bosco’s criteria should not be treated as reflecting obvious, essential, or enduring patient preferences. It therefore seems that employing an automated version of the Spine AI program is unlikely to *meaningfully* reduce the human element in the medical relationship either. In light of this, we believe this case presents an interesting and important opportunity to re-examine the future possibility of kicking humans out of the loop, especially if the opportunity costs of resisting automation are given their proper due.

## Opportunity Costs and Tradeoffs

There is a realistic possibility of automation replacing a significant proportion of a radiologist’s current ordinary workload. Ho et al. (2019) identified numerous activities that stand to be automated, including (1) automated image segmentation, lesion detection, measurement, labeling, and comparisons with historical images; (2) generating radiology reports, particularly with the application of natural language processing and natural language generation; (3) semantic error detection reports; (4) data mining research; and (5) improved business intelligence systems that allow real-time dash-boarding and alert systems, workflow analysis and improvement, outcomes measures and performance assessment (330). While some of the hazards of implementing AI in healthcare have been well established in the literature, discussion of the opportunity costs of *not* capitalizing on the benefits of automation for these novel technologies has received comparatively less attention. Without acknowledgement of the tradeoffs involved, no true assessment of the moral picture can be made. The present case of medical imaging for LSS provides an interesting opportunity to remedy that gap.

As was noted in the Spine AI case, the potential savings in terms of both time and money that stand to be gained from this technology are significant (e.g., ~469,000 minutes (7817 hours) and up to $780,000 SGD each year in Singapore alone). Along with the savings in time and money, there are also meaningful gains to be made in the manpower needed. Again, as noted in the case study, the Spine AI model is able to improve the accuracy of inexperienced trainees, meaning the use of such technologies has the potential to also make each individual radiologist more efficient and effective. The benefits of decreased turnaround time for the reading of scans for LSS should not be understated. In some developing or underdeveloped countries, where medical resources are extremely scarce, the benefits brought about by automated technology are significant. The cost of delaying or foregoing automation is not only economic, but also includes significant health costs and potentially much higher mortality rates. In some situations, patients can wait months for a diagnosis, and this can have a significant impact on their well-being and prognosis, as well as their income and ability to support their families. Automating the time consuming process of image reading would also mean that more people could be effectively served by a smaller cohort of radiologists. Indeed, in some contexts, the backlog of medical images simply means no one ever reads them. In effect, then, the real tradeoff we are presented with when choosing to use or not use an automated image reading system like Spine AI in low-resource settings can be more accurately described as the difference between a patient’s scan being read by a machine or not read *at all.* In fact, in practice, other countries have already chosen to implement automated systems for medical conditions that are more risky than LSS for similar reasons. For instance, in Hong Kong, an automated scan reading system (with no human in the loop and no parallel checks) has been trialed for the detection of intracranial hemorrhage (Abrigo et al. 2023). Because the tradeoffs involved are steeper, the promotion and adoption of automated technology can hold a greater urgency and necessity.

## Criteria for Kicking (Some) Humans Out of the Loop

The norm of keeping humans in the loop is aimed at keeping humans in control over the new technology and making sure that human interests are centered in the process. However, there is no standard sense of what it means to be “in the loop” or indeed of what constitutes a “loop” in the first place. Additionally, it is important to identify which loops are in fact relevant and what kind of role the human should occupy in each (Herman and Pfeiffer, 2023).^3^ In the case of LSS we can break down the tasks involved into the following:

1. Initial patient visit
2. Scanning the patient
3. Segmenting the scan to identify morphology
4. Classifying/grading the degree of stenosis in each location
5. Generating a diagnosis of LSS
6. Making judgements about prognosis
7. Making treatment recommendations.

Though the full “loop” from the patient’s perspective involves 1–7, there is an identifiable and separate radiological “loop” from 2 through 5, and a clinician or surgeon would be in charge of overseeing the other steps. The level of automation we are suggesting in this case would apply to the radiological sub-loop only (e.g., doing away with the radiologist’s role in supervising or performing parallel checks). What criteria, if any, would make that move ethically permissible? To that question, we propose the following:

1. The technology is as effective (or better) than a human at the given task (e.g., error rates are equal to or lower than human experts).
2. The risk to patients (or any humans involved) is low in the event of an error.
3. The wellbeing that is gained by the speed, accuracy, and cost-efficiency of automation is high.

We will explain each criterion in more detail. The Spine AI case presents a good opportunity to examine how these criteria can guide our sense of when it is, or is not, important to keep humans in the loop (and perhaps even more narrowly, when it is important to keep *which* humans in the loop). As mentioned in the case study, the algorithm was able to perform as well or better than a human expert at the given task (measuring the spine to diagnose LSS). The risk to patients in the event of an error on the part of the algorithm is also low (see Discussion section). The Spine AI program is responsible only for making measurements on the MRI scans of the spine, not for any direct treatment of the patient. The riskiness of the condition itself also influences the moral calculus.

While LSS is a potentially debilitating condition, affecting many adults globally and with a considerable impact on livelihood, it is not life-threatening, meaning the stakes in the event of misdiagnosis are lower than if the target condition was a condition like heart failure. Because of the specific features of this kind of case, even if humans are kicked out of the radiological loop, a surgeon will still be available to look at the scan before further treatment decisions are made or implemented, further minimizing risk to patients.

Additionally, other kinds of worries that have been raised regarding the use of medical imaging—like the risk of perpetuating harmful bias—seem minimal or absent given the features of the present case. While some scholars have called attention to various kinds of bias in healthcare (which AI can amplify or enshrine), not all conditions present the same levels of risk when it comes to unjust disparities in care. LSS is not a historically stigmatized condition, given its prevalence across groups, there is no notable association between LSS and negative social stereotypes (like assumptions about laziness). This is good news, since it should mean that there is less risk of the Spine AI program worsening these kinds of worrying biases. That said, racial stereotypes or stigma are far from the only origins for unjust disparities in care (e.g., group differences in consumption of preventive care, and different rates in the uptake of screenings), it nevertheless seems safe to assume that the Spine AI is unlikely to worsen any of these disparities through its use. Given the heterogeneity of the population the AI was trained on, the likelihood of racial bias is low (Hallinan et al. 2021).

A case like that of LSS also presents a strong incentive in terms of the well-being generated through automation’s ability to increase the speed and accuracy of diagnosis while significantly reducing costs. LSS affects people of all races and genders, approximately 103 million people worldwide, equivalent to 11% of the world’s population (Katz et al. 2022). Hence, there is a significant opportunity cost to insisting on keeping humans in the loop for parallel checks on the algorithm’s measurements (increasing cost, less efficiency, more manpower, etc.). As mentioned, the tradeoff is most significant in low and middle income countries (LMICs) where radiologists are often few and far between, and turnaround time for reading the scans can take months, if it happens at all. Automation in this kind of application, therefore, serves the interests of both beneficence and justice. That said, automation of image reading cannot solve all the problems faced by LMICs, which may still struggle with structural limitations like the unavailability of MRI machines to do the scanning in the first place, or surgeons to treat LSS once diagnosed.

Lastly, with a program like Spine AI, the task being automated is one that is mundane and repetitive. This places Spine AI in contrast to more contentious forms of AI (as in the creative realms, like art, writing, etc.), where AI can seem to threaten rather than enable a person’s ability to engage in meaningful human experiences. If such mundane and repetitive tasks are automated, the radiologists themselves do not meaningfully miss out on an opportunity to develop important human qualities or connections. In contrast, their time and attention are liberated and thereby more available for the other dimensions of their profession. Given all these features of the Spine AI case (and by extension any case where these criteria hold), we would argue that it would be morally permissible to kick humans out of the loop.

## Objections

One possible critique of these arguments is that what we are suggesting does not in fact amount to “kicking humans out of the loop.” After all, depending on how one defines a “loop” the label may still be said to apply if a human has the ultimate deciding power. In the case of Spine AI, a human clinician would still inevitably be involved in a later stage of the process (since the taking and the reading of the scan are sandwiched between meetings between the patient and clinician) and would be responsible for other aspects of the diagnostic and treatment process. One may rightly wonder whether this truly is an instance of automation. Essentially, the question comes down to what actually counts as a “loop”—and to which loops are in fact morally important for humans to occupy?

On this note, we are happy to admit that while we may be able to remove some humans from one “loop,” there are still other loops where human involvement remains essential. It was never our intention to argue that humans should be kicked out of all the possible “loops,” nevertheless, we think this is a significant enough step to be worthy of mention. Allowing Spine AI to interpret patient scans without the oversight and monitoring of a radiologist removes the need for parallel checks, which at minimum reduces the number of radiologists needed to serve a given population. Indeed, because of the specifics of this kind of case and the patient pathway involved, even if radiologists are taken out of the loop, a surgeon is present to look at the scan as well. We are not removing all humans from all loops, but removing some humans from one loop. Even if the “loop” that makes up the subject matter of this paper is admittedly a small one, the criteria we have put forth are likely to hold in other instances beyond the present discussion. Such cases are likely to increase in number as the suite of AI technology develops. In line with this, we would argue that it would be permissible to remove the human overseers from those loops as well. Kicking one human radiologist out of one procedural loop may not sound like much, but it is significant since it challenges the assumption that it is something that should in principle never be done.

A second objection that can be foreseen comes from a more pragmatic angle. Knowing that the population is unused to this kind of technology, and that AI has acquired a kind of mystique that leads many to fear it by default—there are practical barriers to implementing even such a minor and innocuous degree of automation. The reasoning may be practical rather than moral in this instance. However, once the reality of the low risks and high rewards are fully communicated, this resistance should wane, and then there would no longer be a reason, moral or practical, to avoid autonomous AIs in these cases which meet the criteria we have laid out. It is important to remember that an insistence on keeping more humans than necessary in the loop can sometimes involve harm in an indirect sense, and this presents us with a trade-off that is, at minimum, important to acknowledge.

## Conclusion

Applications for AI in healthcare continue to develop at a breakneck speed. As they do so, new challenges will emerge, as well as new opportunities. With the discussion of our case, we have highlighted some of the often neglected tradeoffs involved in the development of new ethical norms around novel technologies like this. It is important, as many have highlighted, to ensure to the best of our ability that these new technologies do not expose patients to undue risks, worsen unjust social disparities, or undermine trust in medicine as a whole. But along with those concerns, we must also take care not to lose sight of the very significant benefits that can be provided by these advances and some humans should be kicked out of the loop. It is prudent to use training wheels when learning to ride a bike, but also prudent to anticipate the day that the training wheels can safely come off. To that end, we hope that the criteria laid out here will give some sense of that suitable threshold. When these criteria are met, and there are significant and meaningful gains to be made, we argue that some humans can permissibly be “kicked out of the loop”.

## Footnotes

1 A kappa value is used to measure the agreement between two people (or entities) when categorizing something (in this case LSS). The kappa score expresses how much agreement there is between the two beyond what could be expected by chance alone, with a high kappa score meaning more agreement or overlap.

2 Their findings echo what has been dubbed “the asymmetry of credit and blame” (see Mann et al. (2023) for more on the credit-blame asymmetry in large language models).

3 Hermann and Pfeiffer (2023) identify four different loops in their article: (1) using and assessing AI-output, (2) customizing the AI system, (3) original tasks supported with AI, (4) contextual changes to the system. Writing as they are in the context of organization and AI development, this list is not easily transportable to the Spine AI case, nor is there reason to think this list is exhaustive.
